# Paclitaxel alleviates spinal cord injury via activation of the Wnt/β-catenin signaling pathway

**DOI:** 10.1186/s10020-025-01240-3

**Published:** 2025-05-06

**Authors:** Zhifeng Chen, Da Wo, Celiang Wu, En Ma, Jinhui Peng, Weidong Zhu, Dan-ni Ren

**Affiliations:** 1 Academy of Integrative Medicine, College of Integrative Medicine, Fujian Key Laboratory of Integrative Medicine on Geriatric, Fujian University of Traditional Chinese Medicine, Fuzhou, Fujian China; 2 Department of Orthopedics, Shanghai Changzheng Hospital, Naval Medical University, Shanghai, China

**Keywords:** Paclitaxel, Spinal cord injury, Apoptosis, Wnt/β-catenin signaling pathway

## Abstract

**Background:**

Spinal cord injury (SCI) is a disability that causes severe traumatic damage to the central nervous system, with increasing prevalence worldwide. Paclitaxel (PTX) is a naturally occurring plant metabolite that has been shown to exhibit various neuroprotective effects in the central nervous system, however, the specific mechanisms underlying its protective effects in SCI remain unclear. In this study, we aimed to explore the therapeutic effects of PTX in SCI, as well as elucidate the underlying molecular mechanisms associated with its neuroprotective potential.

**Methods:**

Murine models of spinal cord compression were performed followed by intrathecal administration of corresponding agents for 21 days. Mice were randomly divided into the following four groups: Sham, SCI + Saline, SCI + PTX, and SCI + PTX + XAV939. Recovery of lower limb function and strength, as well as muscular atrophy were examined via multiple scored tests. Degree of neuronal and axonal damage, as well as fibrosis were examined via immunohistochemical staining.

**Results:**

PTX administration significantly improved the recovery of lower limb function and strength, prevented muscular atrophy, as well as decreased the extent of neuronal and axonal death following SCI surgery. PTX also robustly activated the Wnt/β-catenin protein signaling pathway that played a key role in its therapeutic effects. Co-administration with a Wnt/β-catenin pathway inhibitor - XAV939, significantly abolished the beneficial effects of PTX after SCI.

**Conclusion:**

This study provides important new mechanistic insight on the beneficial effects of PTX in protecting against spinal cord injury, as well as the experimental basis for its potential therapeutic use.

**Supplementary Information:**

The online version contains supplementary material available at 10.1186/s10020-025-01240-3.

## Introduction

Spinal cord injury (SCI) is a common disability that is caused by vertebral fractures or dislocations to the spinal cord, which leads to motor, sensory, and autonomic dysfunction below the level of injury (Huang et al. 2020; Ju et al. 2014). Due to the rapid development of industry and transportation, the incidence of SCI has increased rapidly worldwide, which poses a significant burden to both individuals and their families (Karsy and Hawryluk 2019; Zhou et al. 2024a, 2024b). Therefore, finding effective therapeutic drugs that can alleviate or prevent injury following SCI is of great clinical significance.

Paclitaxel (PTX) is a natural metabolite that is primarily produced by plants belonging to the genus Taxus. Modern pharmacological studies have shown that these plants possess numerous pharmacological activities, such as promoting tubulin polymerization, anti-fibrotic, anti-inflammatory, anti-bacterial, anti-convulsant, and anti-oxidative effects (Ahmed Khalil et al. 2022; Gallego-Jara et al. 2020). In the central nervous system (CNS), axons typically do not regenerate after injury, and instead form characteristic swellings at their tips known as retraction bulbs, which contain a disorganized microtubule network (Kulkarni et al. 2022). PTX can stabilize microtubule formation by aggregating and organizing the disordered microtubules into an organized arrangement. This stabilization prevents the rapid depolymerization of microtubules and the formation of retraction bulbs after injury, thereby supporting and maintaining microtubule stability and axonal extension (Ertürk et al. 2007; Roman et al. 2016). PTX administration at the site of traumatic SCI has been shown to be effective in reducing the formation of glial scars, while enhancing functional recovery and promoting the growth of serotonergic axons after both hemisection and moderate spinal cord contusion injuries (Hellal et al. 2011). Numerous studies have demonstrated that PTX can also prevent axonal retraction and promote axonal growth and regeneration of sensory axons with growth potential at the lesion site. Moreover, PTX reduces leukocyte infiltration and migration, decreases fibrotic scar formation, and ultimately facilitates the recovery of function and strength following spinal cord injury (Hellal et al. 2011; Popovich et al. 2014; Roman et al. 2016). However, the specific underlying mechanism by which PTX promotes functional and strength recovery after SCI remains unclear.

Wnt/β-catenin pathway is most commonly associated with cell proliferation and differentiation (Austin et al. 2021; Ma et al. 2023). Recent studies have suggested that activation of the Wnt/β-catenin pathway plays a key role in neuron survival and regeneration following nerve injury (Cui et al. 2019; Marchetti 2018; Zhang et al. 2020). Various drugs such as rapamycin, methylprednisolone, and electroacupuncture have been shown to exhibit beneficial effects in protecting against SCI via activating Wnt/β-catenin signaling pathway (Gao et al. 2015; Liu et al. 2023a, 2023b; Lu et al. 2016). Wnt/β-catenin pathway is also critically involved in the regulation of the inflammatory response and cell apoptosis after SCI (Ding and Chen 2024; Gao et al. 2024). However, the effect of PTX on regulating Wnt/β-catenin pathway following SCI has not been explored. Changes in the Wnt/β-catenin pathway can alter the sensitivity of various tumor cells to paclitaxel, including bladder cancer, esophageal squamous cell carcinoma, and breast cancer (Jiménez-Guerrero et al. 2021; Song et al. 2024; Zhou et al. 2024a, 2024b). Our current study aimed to elucidate the therapeutical effects and mechanisms of PTX in protecting against SCI via the regulation of Wnt/β-catenin signaling pathway.

## Materials and methods

### Drugs and Reagents

Paclitaxel (PTX) was purchased from MedChemExpress (MCE, China), CAS number: 33069-62-4, purity: 99.96%. PTX was dissolved in dimethyl sulfoxide (DMSO) to prepare a 117 mM stock solution, aliquoted, and stored at -80°C for further use. For *in vivo* experiments, PTX and XAV939 were administered at dosages of 36 ng/day/mice, and 0.56 mg/kg/mice, respectively. PTX injection dosage and method of injection was based on the effective dose as previously described (Hellal et al. 2011; Popovich et al. 2014; Zhilai et al. 2012). This final dosage used in our study was calculated based on the equivalent dose conversion formula between rats and mice.

### Animal studies

All procedures were performed in accordance with the Guide for the Care and Use of Laboratory Animals published by the National Institutes of Health, comply with the ARRIVE guidelines and approved by the Experimental Animal Care and Use Committee of Fujian University of Traditional Chinese Medicine (Approval No.: FJTCM IACUC 2023034). Female C57BL/6 mice (6-8 weeks old, 17-21 g) were obtained from Shanghai SLAC Laboratory Animal Co., Ltd. (Shanghai, China). Mice were randomly divided into four groups: Sham group (n=6), SCI + Saline group (n=8), SCI + PTX group (n=8), and SCI + PTX + XAV939 group (n=8). Mice were anesthetized with an intraperitoneal injection of 0.2% sodium pentobarbital. After anesthesia and disinfection, a midline incision of approximately 1.5 cm was made over the thoracic vertebrae, and a laminectomy was performed at T9-T12. SCI was performed according to standard protocol. Briefly, mice were anesthetized and a midline incision of approximately 1.5 cm was made over the thoracic vertebrae, and a laminectomy was performed at T9-T12. Surgical forceps with tip width of 0.1 mm (RWD, China, F11027-13) were inserted around both sides of the spinal cord. To ensure the inclusion of the entire spinal cord width, forceps were gently scraped along the ventral side of the spinal canal. Subsequently, the spinal cord was completely clamped with a firm compression force using forceps for 2 seconds to produce a uniformly thin injury site. In the sham group, a laminectomy was performed without the subsequent compression injury. After mice regain consciousness, mice were assessed via the Basso Mouse Scale (BMS). Mice that receive a score of 0 were considered a successful model for SCI, while those that did not meet this criterion were excluded from the study. Immediately after surgery, mice were administered with PTX via intrathecal injection (i.t.). PTX or XAV939 were administered intrathecally (i.t.) daily for 21 days post-surgery. Sham and SCI groups received equal volumes of saline injections. Penicillin was administered via intramuscular injection for the first three days to prevent infection. Mice bladders were manually expressed via perineal stimulation once daily to aid urination until recovery of bladder function.

### Locomotion recovery assessment

Mice were evaluated at different time points (postoperative days 1, 3, 7, 10, 14, and 21) after SCI using the Basso Mouse Scale (BMS) and toe spread scores to assess the degree of hindlimb functional recovery. BMS is a scoring method proposed by Basso et al. to describe the degree of hind limb behavioral recovery in mice after SCI (Basso et al. 2006). The scoring scale primarily evaluates ankle joint movement, paw placement, paw posture, trunk stability, tail position, and tail movement, ranging from 0 points (no observable locomotor movements) to 9 points (normal locomotor movements). A higher score indicates better recovery of hind limb motor function. In particular, scores of 0 to 2 assess ankle joint movement, scores of 3 to 4 evaluate hind limb support and forward stepping, scores of 5 to 8 assess hind paw placement and coordination of forelimbs and hind limbs during stepping, and a score of 9 represents normal movement. Toe spreading is a reflex elicited by lifting the mouse, causing its toes to spread. The mouse is placed on a table, with one hand holding its body and lifting its legs to allow free hanging. The hind limb toe spreading score is graded as follows: 0 points, no toe spreading; 1 point, relaxed toes with minimal spreading; 2 points, partial toe spreading; 3 points, normal full toe spreading. The maximum slipping angle in the incline plane test was measured in mice at postoperative days 6, 9, 12, and 20 after SCI to quantify and compare differences in hindlimb muscle strength among the groups. The incline plane test and grip strength test are methods used to assess limb strength and body stability in mice after SCI. In the incline plane test, the mouse is placed on a flat but non-slippery inclined plane, and the angle between the plane and the horizontal surface is gradually increased until the mouse slides down. The maximum angle at which the mouse can maintain its position for at least 5 seconds is recorded. Each mouse is measured three times, and the average value is recorded. The grip strength test was conducted in mice at postoperative days 7, 10, 14, and 21 after SCI, and the specific steps are as follows: the mouse is held with the right hand, allowing its body to stretch naturally with its limbs extended. After an initial adaptation grip, and once the readings stabilize, 10 values are recorded for each mouse. The average grip strength is then calculated and recorded.

### Hematoxylin-eosin (HE) and Masson's trichrome staining

Histological staining was performed according to standard protocol. Briefly, mouse spinal cords were fixed in 4% paraformaldehyde for 24 hours and washed with tap water overnight prior to dehydration using an ethanol gradient. Subsequently, tissues were cleared in xylene, embedded in paraffin, and sectioned into 8 μm thick longitudinal slices. For HE staining, the sections were stained with HE reagent according to the manufacturer's instructions (Solarbio, China, G1120) and observed under an optical microscope (Leica, Germany). Masson's trichrome staining was used to visualize the distribution and extent of spinal cord glial scarring. Under the microscope, spinal cord sections were observed, where the fibrotic areas with collagen deposition were stained blue, normal spinal cord tissue was stained red, and cell nuclei appeared blue-black.

Image quantifications including lesion area and percentage of collagen fibers were performed using ImageJ software, as detailed below. The injury area was marked to calculate the size of the injury. The spinal cord lesion area was calculated as (lesion area of the spinal cord / total spinal cord area) × 100%. For proportion of collagen fibers, whole-slide images were processed with color deconvolution to extract the blue-stained collagen elements. Next, manual delineation of Regions of Interest (ROIs) covering the entire tissue area was performed after excluding artifacts and non-tissue regions. A threshold-based segmentation method (Hue-Saturation-Brightness mode: 180-255 Hue, 0-255 Saturation, 0-220 Brightness) was utilized to distinguish collagen fibers from background and cellular components. The fibrotic area ratio (%) was then calculated as (collagen-positive pixels after thresholding / total tissue pixels) × 100. Vascular structures and tissue folds were systematically omitted using the "Freehand Selection" tool to reduce variability. All threshold configurations were verified against control samples and consistently maintained across comparative groups. The number of stained slides and sections used were as follows: Sham group (n=3), SCI + Saline group (n=4), SCI + PTX group (n=4).

### Luxol fast blue (LFB) staining

For LFB staining, the sections were stained overnight in 0.1 % LFB at 60 ◦C (Solarbio, China, G3245). Thereafter, the sections were washed in double-distilled water, and then incubated briefly in 0.05 % lithium carbonate, and finally observed using optical microscope (Leica, Germany).

### Nissl and immunofluorescence Staining

Fresh spinal cord tissue was dehydrated with sucrose and embedded in Tissue Tek OCT compound, followed by rapid freezing on dry ice. The tissue was then sectioned into 25 μm thick slices onto charged hydrophilic microscope slides. For Nissl staining, the sections were stained using Nissl staining reagents according to the manufacturer's instructions (Beyotime, China, C01117), and observed using an optical microscope (Leica, Germany). Under the optical microscope, cells positive for Nissl staining exhibit a mottled blue-purple coloration. Observations and photographic image capture were conducted at 100x magnification. The numbers of Nissl-stained positive cells in the field of view for each group of sections were counted using ImageJ software.

Immunofluorescence staining was performed according to standard procedures. Samples were washed with PBS, fixed in 4% paraformaldehyde for 30 minutes, permeabilized using 0.25% Triton X-100 in PBST (0.5% Tween-20 in PBS), blocked using 5% bovine serum albumin (BSA), and incubated with primary antibodies overnight at 4°C. Subsequently, samples were incubated with Alexa Fluor-conjugated secondary antibodies (Abcam, USA) and mounted in anti-fade mounting medium containing DAPI. Immunofluorescence images were captured using an inverted fluorescence microscope (Leica, Germany). Fluorescence intensity was calculated using ImageJ software. Briefly, images were converted to 16-bit grayscale images and cellular axons were outlined using threshold-based segmentation or manual tracing. For multi-channel images, color deconvolution was used to isolate signals. Background subtraction was performed via "Subtract Background" algorithm with a 50-pixel radius and removal of saturated pixels (intensity ≥4095). The number of stained slides and sections used were as follows: Sham group (n=4), SCI + Saline group (n=4), SCI + PTX group (n=4).

### Western Blot Analysis

Western blot analysis was performed according to standard protocol. Briefly, following mouse sacrifice, spinal cords and muscles were carefully harvested and snap-frozen in liquid nitrogen. Total proteins were extracted using RIPA lysis buffer (Beyotime Biotech, China) via sonication. Nuclear and cytoplasmic proteins were extracted with nucleoprotein extraction kit (Sangon Biotech, China). Equal concentrations of proteins were loaded onto SDS-PAGE gel for electrophoresis, transferred onto 0.22 µm polyvinylidene difluoride (PVDF, Merck Millipore, MA, USA) membrane, blocked with 5% nonfat dried milk, then incubated with the indicated primary antibodies overnight at 4 ^◦^C. Subsequently, membranes were incubated with the secondary antibodies and the resulting protein bands were detected via chemiluminescence. Quantification of blots was performed using ImageJ software. Briefly, images were first converted to 8-bit grayscale and rectangular regions of interest (ROIs) were then drawn around each target band with identical dimensions. Background subtraction was carried out by measuring the adjacent non-band areas and using the "Rolling Ball" algorithm (radius = 50 pixels). Integrated density values (IDV) were recorded for each band after adjusting the threshold to remove nonspecific signals. Subsequently, normalization was performed against the corresponding loading controls for each band.

The following antibodies were used: GAP43 (ab75810) was purchased from Abcam, USA. NeuN (26975-1-AP), NF200 (18934-1-AP), Bcl2 (26593-1-AP), c-Myc (10828-1-AP) and β-actin (66009-1-Ig) were purchased from Proteintech, USA. Myosin (M8421) was purchased from Sigma-Aldrich, USA. Phospho-β-Catenin (9561S), β-catenin (8480S), Bax (14796S), Caspase-3 (9662S) and TBP (44059S) were purchased from Cell Signaling Technology, USA. Goat anti-Mouse IgG secondary antibody (6229) and Goat anti-Rabbit IgG secondary antibody (8715) were purchased from Signalway Antibody, USA.

### TOPFlash reporter assay

TOPFlash reporter gene assays (Vazyme, China, DD1205) were performed according to the manufacturer’s instructions for the examination of Wnt/β-catenin signaling pathway activation. Briefly, HEK293 cells (2.5 × 10^4^ cells/well) were seeded on 48-well plates and cultured in a 37 ^◦^C incubator supplemented with 5% CO_2_. Cells were co-transfected with Wnt3a (2 ng) and TOPFlash (Beyotime, China, D2501) for 48 h. At 24 h post-transfection, cells were treated with PTX for 24 h, after which cell lysates were harvested and reporter activities were assayed using a Dual-Luciferase Reporter Assay System. All data were normalized by Renilla luciferase activity.

### Statistical analysis

All statistical analyses were performed using SPSS 22.0 software, and data were presented as the mean ± standard error of mean (SEM). One-way analysis of variance (ANOVA) with Fisher’s LSD post-hoc analysis was used for comparisons between three or more groups, and independent sample t-test was used for comparisons between two groups. P < 0.05 was considered statistically significant.

## Results

### PTX improves recovery of locomotor function following SCI

**Fig. 1:**
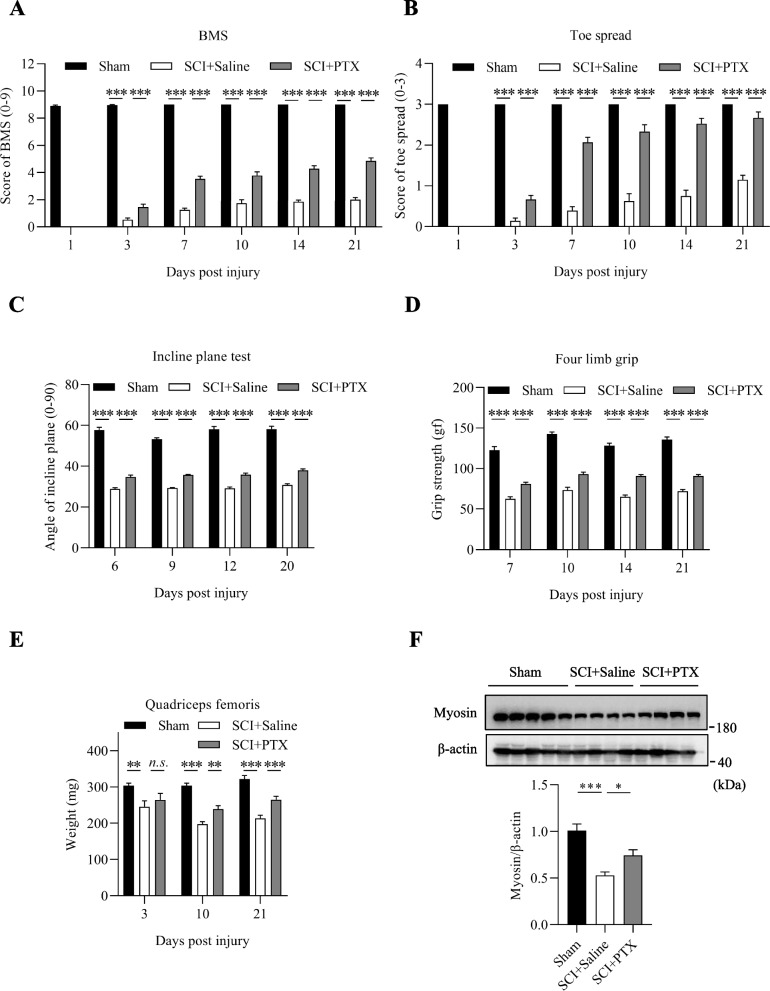
PTX improves recovery of locomotor function, promotes recovery of limb strength recovery, and prevents lower limb muscle atrophy following SCI. (A) Postoperative day 21 BMS scores were assessed in mice. (B) Postoperative day 21 toe extension scores were evaluated in mice. (C) Postoperative day 21 inclined plane experiments were conducted in mice. (D) Postoperative day 21 grip strength experiments were performed in mice. (E) Quadriceps muscle weight of each group of mice on postoperative day 3, 10 and 21. (F) Representative immunoblots (top) and densitometry analysis (bottom) of myosin expression in the spinal cords of mice on postoperative day 21.β-actin was used as the loading control. Data are presented as mean ± sem, n.s., not significant, *P < 0.05, **P < 0.01, ***P < 0.001. n = 4 or more in each group

In order to assess the potential beneficial effects of PTX in promoting recovery of locomotive function, we utilized the Basso Mouse Scale (BMS) and toe extension measurements for scoring mice that underwent spinal cord injury (SCI) surgery. Initial scores for BMS and toe spread were recorded prior to surgery, and post-surgery, all mice were confirmed to score 0 for all parameters. Notably, the PTX group scored significantly higher on the BMS scale compared to the saline group from postoperative day 3 (Fig. 1A), which became progressively improved over time through to postoperative day 21 (Fig. 1A). In addition, the PTX group exhibited significantly higher toe extension scores compared to the saline group from postoperative day 3 (Fig. 1B), and this trend became more obvious and almost matched scores of sham-operated mice from postoperative day 7 onwards (Fig. 1B). Taken together, these results demonstrated that PTX intervention robustly promoted the recovery of lower limb motor function following SCI surgery.

### PTX promotes recovery of limb strength following SCI

To examine the effects of PTX on the recovery of limb strength in mice following SCI surgery, we further performed incline plane test and grip strength experiments. Mice with greater limb strength can resist falling from greater slide angles in the incline plane test, with mice in the sham-operated group able to resist slide angles of above 50°. Mice that underwent SCI surgery and administered with saline exhibited significant decreases in slide angles at all time intervals (Fig. 1C). Notably, mice administered with PTX showed significant improvements in the incline plane angles compared to those administered with saline (Fig. 1C), indicative of increased limb strength following SCI surgery.

Assessment of grip strength was determined by the average of both forelimb and hindlimb, i.e. four limb grip strength, with sham-operated mice having grip strengths of approximately 120 gf. Mice that underwent SCI surgery and administered with saline had significantly reduced grip strengths at all time intervals (Fig. 1D). Notably, mice administered with PTX showed significant improvements in grip strength compared to the saline group, which became progressively stronger over time (Fig. 1D). Taken together, these results further demonstrate that PTX significantly promotes the recovery of limb strength following SCI surgery.

### PTX prevents lower limb muscle atrophy after SCI

A major outcome of SCI is muscular atrophy of the quadriceps muscle due to decreased mobility of the lower limbs. Examining changes in lower limb muscle mass and strength may provide an indication on postoperative recovery following SCI. Hence, we measured the changes in the muscle mass of mouse quadriceps muscles at postoperative days 3 and 10 after SCI surgery. Although there were no significant differences in lower limb muscle mass between the PTX and saline groups on postoperative day 3 (Figs. 1E), mice administered with PTX had significantly greater recovery of quadriceps muscle mass by postoperative days 10 and 21 (Figs. 1E).

Furthermore, we examined the protein levels of myosin in the lower limb muscles following SCI, which is representative of myofibril level and muscle mass. Notably, SCI surgery resulted in significant reduction in myosin levels in the quadriceps, but was markedly attenuated in mice treated with PTX (Fig. 1F). Taken together, these findings demonstrate that PTX alleviates muscle atrophy caused by SCI-induced lower limb paralysis and facilitates postoperative recovery.

### PTX improves the morphology of damaged spinal cord tissue

**Fig. 2:**
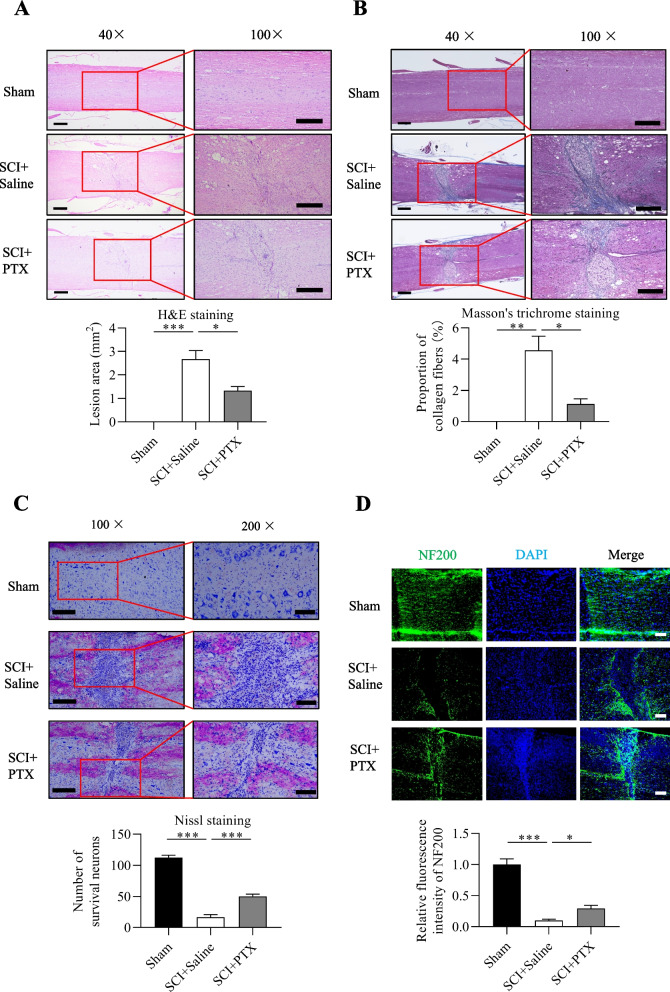
PTX improves the morphology of damaged spinal cord tissue and promotes neuronal and axonal survival after SCI. (A) HE staining images of spinal cords and SCI areas in each group of mice on postoperative day 21. (B) Masson staining images of spinal cords and glial scar areas in each group of mice on postoperative day 21. (C) Nissl staining images of spinal cords and number of Nissl-stained positive cells in each group of mice on postoperative day 21. (D) NF200 fluorescent staining and analysis of NF200 fluorescence intensity in each group on postoperative day 21 (100×), green: NF200, and blue: DAPI. Data are presented as mean ± sem, *P<0.05, **P<0.01, ***P<0.001. n=3 or more in each group. 40× scale bar, 500 μm, 100× scale bar, 100 μm and 200× scale bar, 100 μm

We next examined the morphology of spinal cord tissues at postoperative day 21 following SCI surgery using Hematoxylin and Eosin (HE) staining. In contrast to sham-operated mice that had intact spinal cord structure, mice that underwent SCI exhibited disrupted tissue structure and formation of necrotic cavities (Fig. 2A). Notably, PTX-administered mice exhibited noticeable improvements in tissue structure compared to saline-administered mice, with a significant reduction in necrotic cavity formation (Fig. 2A). Furthermore, the PTX group had significantly reduced cross-sectional area of spinal cord tissue damage compared to the saline group (Fig. 2A), demonstrating that PTX significantly prevented spinal cord tissue destruction following SCI surgery.

Another pathophysiological defect caused by SCI is the formation of glial scar tissue, which impedes the growth and connectivity of axons in the injured area. To further investigate the effect of PTX on glial scar formation after SCI, we utilized Masson's trichrome staining to observe the morphological changes in the injured spinal cord at postoperative day 21. Mice that underwent SCI exhibited obvious formation of glial scar tissues in the injured spinal cord compared to sham-operated mice, which was significantly reduced in mice administered with PTX (Fig. 2B). These results further demonstrate that PTX can effectively inhibit glial scar tissue formation following SCI surgery. We further examined the extent of white matter sparing post-SCI via LFB staining, which showed that following SCI, there was severe myelin sheath destruction with loss of the myelin sheath compared to sham-operated mice (Supplementary Fig. 1). In contrast, mice that received PTX treatment significantly ameliorated these defects. These results demonstrate that PTX can also improve functional and locomotor recovery in the alleviation of spinal cord injury.

### PTX increases neuronal and axonal survival after SCI

We further examined the morphological changes and number of surviving neurons in the spinal cord following SCI surgery via Nissl staining, which reflects the metabolic activity and functional state of neurons. Compared to sham-operated mice that had intact neurons with clear Nissl bodies in the cytoplasm, mice that underwent SCI exhibited markedly abnormal morphology, with large cavities, damaged cellular structures and unclear Nissl bodies (Fig. 2C). Notably, mice administered with PTX exhibited markedly improved spinal cord tissue morphology, with relatively intact axon structure, small cavities and greater number of Nissl bodies indicative of normal or surviving neurons (Fig. 2C). These results indicate that PTX significantly mitigated neuronal injury following SCI.

In addition, we performed immunofluorescence staining using the axon specific marker NF200, in order to visually observe the number and distribution of axons in the spinal cords of mice following SCI surgery. Mice that underwent SCI had significantly reduced staining for NF200 and damaged axonal structure compared to sham-operated mice, which was markedly rescued in PTX-treated mice (Fig. 2D). Taken together, these results demonstrate that PTX effectively protects against axonal loss and neuronal injury after SCI.

### PTX reduces neuronal death and promotes axonal regeneration after SCI

**Fig. 3:**
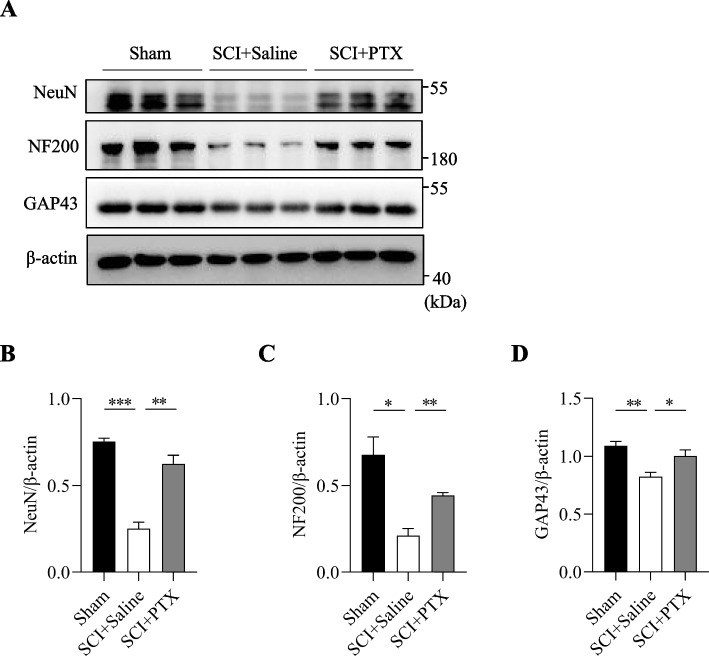
PTX reduces neuronal death and promotes axonal regeneration after SCI. (A-D) Representative immunoblots and densitometry analysis of NeuN, NF200 and GAP43 expression in the spinal cords of mice on postoperative day 21. β-actin was used as the loading control. Data are presented as mean ± sem, *P<0.05, **P<0.01, ***P<0.001. n=3 in each group

Furthermore, we examined the levels of neural marker-related protein NeuN and NF200, along with the regenerating axon marker GAP43, in the spinal cord following SCI via Western blot analysis. Notably, NeuN, NF200, and GAP43 levels were significantly reduced in the spinal cord tissues at postoperative day 21 following SCI, but were markedly rescued by PTX treatment (Fig. 3A-D). These findings indicate that PTX exhibits strong neuroprotective benefits to the injured spinal cord following SCI, by decreasing neuronal death and promoting axonal regeneration.

### PTX activates the Wnt/β-catenin signaling pathway

**Fig. 4:**
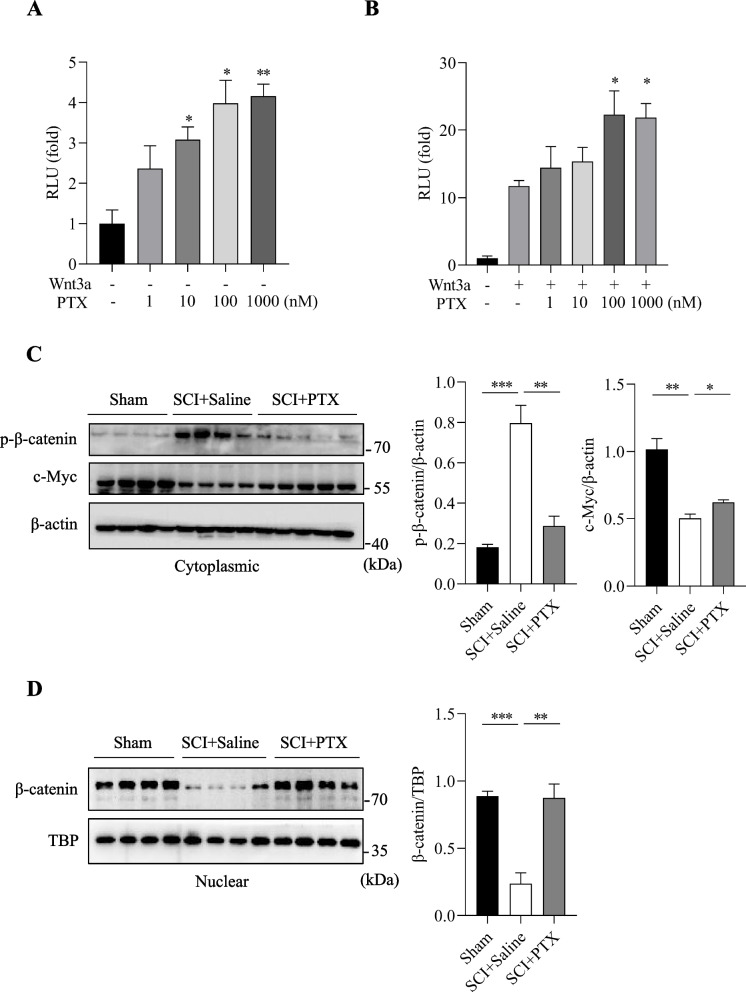
PTX activates the Wnt/β-catenin signaling pathway. (A) The effect of baseline PTX levels on the Wnt/β-catenin signaling pathway. (B) The effect of PTX in conjunction with Wnt3a on the Wnt/β-catenin signaling pathway, Wnt3a was transfected with TOPFlash in HEK293 cells for 48 h, PTX or PBS were directly added to the transfected cells at varying concentrations, as indicated. RLU, relative light units. (C and D) Representative immunoblots and densitometry analysis showing cytoplasmic p-β-catenin, c-Myc, and nuclear β-catenin in the spinal cords of mice on postoperative day 21. β-actin and TBP was used as the loading control respectively. Data are presented as mean ± sem, *P<0.05, **P<0.01, ***P<0.001. n=3 or more in each group

There is accumulating evidence that suggests Wnt/β-catenin signalling pathway plays a central role in spinal cord injury. Hence, we further examined whether the underlying mechanism of PTX in protecting against SCI may be related to Wnt/β-catenin pathway activation. TOPFlash reporter gene assays showed that the Wnt/β-catenin signaling pathway was significantly activated after PTX treatment, in a dose-dependent manner (Fig. 4A). Moreover, we overexpressed Wnt via transfection of Wnt3a plasmid, and PTX also further promoted Wnt ligand Wnt3a-induced activation of the Wnt/β-catenin signaling pathway (Fig. 4B), also in a dose-dependent manner. These results demonstrate that PTX is a robust agent in promoting the activation of Wnt/β-catenin signalling pathway.

Next, we examined the protein levels of β-catenin and c-Myc in the spinal cord tissue following SCI surgery and PTX intervention. The levels of nuclear β-catenin and cytoplasmic c-Myc were markedly decreased following SCI surgery but were significantly rescued in mice treated with PTX (Fig. 4C and D). Supporting this result, the levels of p-β-catenin – indicative of cytoplasmic degradation, became markedly upregulated in SCI mice, which was attenuated in PTX-administered mice (Fig. 4C). Taken together, these findings suggest that PTX intervention results in sustained elevations of β-catenin and c-Myc in the spinal cord tissues following SCI, thereby protecting against SCI via robustly activating the Wnt/β-catenin signaling pathway.

### Wnt pathway inhibitor XAV939 abolishes the therapeutic effects of PTX after SCI

**Fig. 5:**
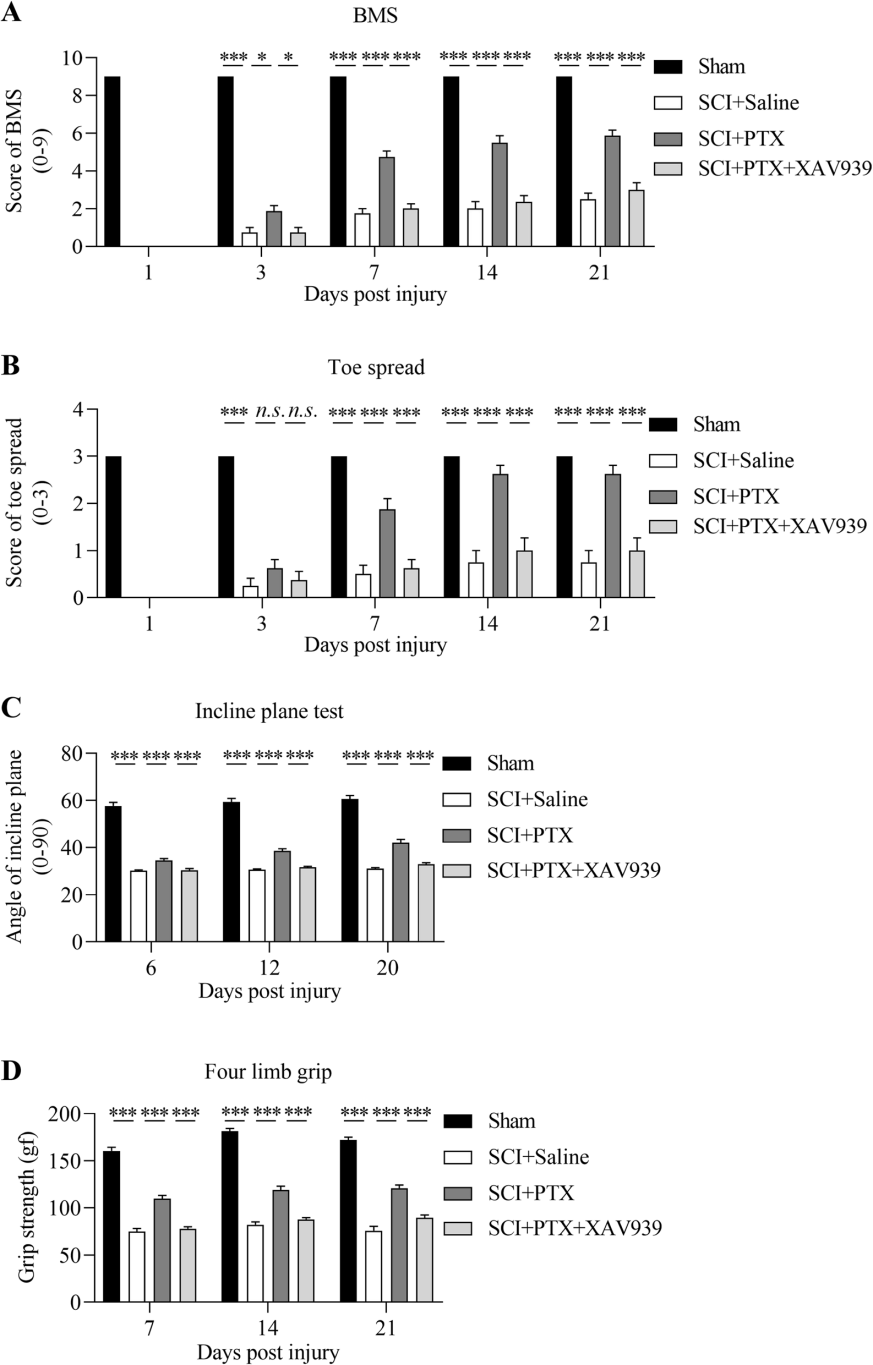
Wnt pathway inhibitor XAV939 abolishes the therapeutic effects of PTX after SCI. (A) BMS scores of each group of mice on postoperative day 21. (B) Toe extension scores of each group of mice on postoperative day 21. (C) Inclined plane test results of each group of mice on postoperative day 21. (D) Grip strength test results of each group of mice on postoperative day 21. Data are presented as mean ± sem, n.s., not significant, *P<0.05, **P<0.01, ***P<0.001. n=6 or more in each group

In order to verify that the beneficial effects of PTX in spinal cord injury were specifically due to the activation of Wnt/β-catenin signaling, we further administered mice with XAV939 (Wnt/β-catenin pathway inhibitor) following SCI surgery. Notably, the therapeutic effects of PTX on locomotor function including BMS and toe extension scores were significantly prevented in mice co-administered with XAV939 (Fig. 5A and B). Furthermore, the effects of PTX on recovery of limb strength was also significantly reduced following XAV939 administration, in both inclined plane and grip strength tests (Fig. 5C and D). These findings further support that the effects of PTX in promoting motor function following SCI were via activation of the Wnt/β-catenin pathway.

Similarly, the protective effect of PTX on lower limb muscle atrophy following SCI was significantly decreased when co-administered with Wnt inhibitor XAV939 (Supplementary Figure 2A). Furthermore, body weight that is indicative of an overall physiological status, exhibited a similar trend from postoperative days 7 to 21 (Supplementary Figure 2B). In addition, WB analysis showed that although mice administered with PTX significantly rescued the SCI-induced reductions of NeuN, NF200, and GAP43, mice that were co-administered with XAV939 completely prevented this neuroprotective ability of PTX (Supplementary Figure 2C-F). These findings demonstrate that the effects of PTX in preventing lower limb muscle atrophy following SCI were via activation of the Wnt/β-catenin signaling pathway.

### PTX inhibits cell apoptosis following SCI via regulating Wnt/β-catenin pathway

**Fig. 6:**
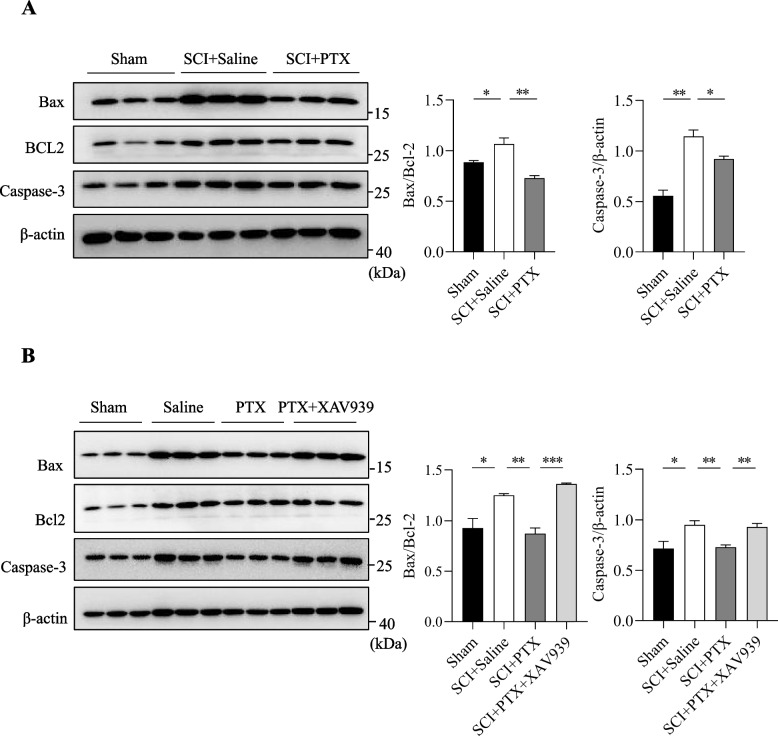
PTX inhibits neuron cell apoptosis following SCI via regulating Wnt/β-catenin pathway. (A) Representative immunoblots (left) and densitometry analysis (right) of Bax, Bcl-2, and Caspase-3 expression in the spinal cords of mice on postoperative day 21. (B) Representative immunoblots (left) and densitometry analysis (right) of Bax, Bcl-2, and Caspase-3 expression in the spinal cords of mice co-administered with PTX and XAV939 on postoperative day 21. Data are presented as mean ± sem, *P<0.05, **P<0.01, ***P<0.001. n=3 in each group

Wnt/β-catenin pathway is also critically involved in cell apoptosis, a key regulator of neurological function following SCI. Indeed, our results demonstrated that levels of caspase-3 and Bax/Bcl-2 ratio indicative of cell apoptosis, were significantly increased in the spinal cord tissues following SCI surgery (Fig. 6A). Interestingly, mice administered with PTX significantly attenuated these SCI-induced increase in cell apoptosis in the spinal cord tissues, suggesting that PTX can effectively inhibit apoptosis occurrence following SCI (Fig. 6A).

Notably, mice co-administered with XAV939 prevented the ability of PTX in reducing the levels of Bax/Bcl-2 and caspase-3 (Fig. 6B) in the spinal cord following SCI surgery, further demonstrating that PTX promotes SCI recovery by inhibiting neuronal apoptosis via activating the Wnt/β-catenin signaling pathway.

## Discussion

Recent studies have shown that activation of the Wnt/β-catenin signaling pathway aids in the functional recovery of the central nervous system, improves neuron survival, promotes axon regeneration, and alleviates neuropathic pain (Onishi et al. 2014; Yang et al. 2013). Wnt/β-catenin signaling activation also plays a key role in promoting functional recovery and neuronal survival following SCI (He et al. 2023; Qi et al. 2022). Wnt/β-catenin signaling activation has also been shown to enhance endogenous neurogenesis and inhibit neuronal apoptosis in rats with ischemic stroke (Zhang et al. 2022). In our current study, we showed that PTX activates the Wnt/β-catenin signaling pathway in a dose-dependent manner in HEK293 cells via TOPFLASH reporter gene assay, while co-administration with Wnt3a further augmented the activation of the Wnt/β-catenin signaling pathway. This demonstrates that PTX can directly activate the Wnt/β-catenin signaling pathway in the absence of Wnt ligands but also enhance the effect of Wnt3a ligand in activating Wnt/β-catenin signaling pathway. Further, the effects of PTX were prevented following treatment with the specific Wnt/β-catenin signaling inhibitor XAV939, which supports the fact that PTX exerts its neuroprotective effects in SCI via positive regulation of the Wnt/β-catenin signaling pathway. These finding reveal the potential underlying mechanism of PTX’s strong therapeutic effects in the treatment of SCI.

One of the main factors that influence the degree of injury after SCI is the death of residual cells around the injury site surrounding the spinal cord (Shi et al. 2021; Sterner and Sterner 2022). Hence, decreasing the degree of neuronal apoptosis following SCI has showed positive outcomes in promoting recovery of the injury site (Abbaszadeh et al. 2020; Liang et al. 2022). Caspase-3 is one of the key components of the apoptotic cascade during SCI, which primarily occurs in neuronal cells (Fang et al. 2021; Han et al. 2022). Other key members include the Bcl-2 family of anti-apoptotic proteins, as well as the BAX family of pro-apoptotic proteins (Spitz and Gavathiotis 2022; Xie et al. 2024). Excessive apoptosis eventually leads to neuronal necrosis and widespread death of neurons. Therefore, drugs that can effectively reduce neuronal apoptosis is crucial for promoting SCI recovery. Multiple studies have revealed that activating the Wnt/β-catenin signaling pathway after SCI can effectively inhibit neuronal apoptosis and promote neuronal regeneration(Briona et al. 2015; Li et al. 2019a; Zhou et al. 2020). In the present study, we revealed for the first time that the natural plant metabolite PTX also exhibited robust effects in preventing neuronal apoptosis, thereby promoting recovery of neurons and axons following SCI surgery. Importantly, we demonstrated that these neuroprotective effects of PTX was via robust activation of the Wnt/β-catenin signaling pathway, whereby co-administration of the Wnt/β-catenin pathway inhibitor XAV939 completely prevented the therapeutic benefits of PTX. These results elucidate the underlying neuroprotective mechanism of PTX in the treatment of SCI.

Previous studies regarding the dosage effects of PTX showed that although low concentrations of PTX do not block microtubule dynamics, it can still inhibit the speed and extent of microtubule contraction, in that PTX can still promote the polymerization of microtubules and promote neuronal axon growth. In contrast, high concentrations of PTX inhibit microtubule dynamics, thereby suppressing axon elongation and causing toxicity to cells (Ertürk et al. 2007). Therefore, the dosage of PTX used for the treatment of SCI is much lower than the corresponding dosage used in cancer treatment.

Of note, among cancer patients receiving PTX treatment, approximately 60-70% of patients develop PTX-induced peripheral neuropathy (Seretny et al. 2014). PTX initially acts on the microtubules of sensory neuron axons, leading to changes in intracellular calcium regulation, axonal transport, mitochondrial function, and neuropeptide secretion. Therefore, the interactions between sensory neurons in the peripheral nervous system and the surrounding cells may lead to the development of neuropathic pain (Li et al. 2019b; Sekiguchi et al. 2018). Therefore, determining the effective dosage and treatment plan for PTX in clinical practice is crucial, in order to provide the most effective dosage for treatment of spinal cord injury, while limiting the potential side effects associated with high PTX dose.

PTX has previously demonstrated microtubule-stabilizing effects, preventing characteristic swellings containing microtubule networks in damaged axons in the central nervous system (Xu et al. 2024; Zhao et al. 2022). PTX can also promote axonal growth capacity of neurons under inhibitory conditions (Ertürk et al. 2007; Liu et al. 2023a). PTX stabilizes microtubule formation by aggregating and stabilizing disorganized microtubules into orderly structures, preventing rapid microtubule depolymerization after injury, thereby maintaining microtubule stability and axonal extension. Furthermore, microtubule stabilization is known to reduce fibrotic scar formation and enhance axonal growth capacity following injury (Roman et al. 2016; Ruschel and Bradke 2018; Xu et al. 2022). These studies further support the results of our study, which demonstrated that PTX treatment robustly promoted neuronal and axonal survival following SCI injury, leading to the greater recovery of limb strength. There are several limitations regarding our current study. Firstly, we utilized the murine model of spinal cord compression injury, which results in more severe injuries compared to standard impact-associated spinal cord injuries that are common in clinical cases such as car accidents or falls from heights. Therefore, for better comparative analysis, it may be useful to create new animal research models based on impact-associated injuries, which may be more clinically impactful. Secondly, electromyography assessment to detect the neuromuscular conduction of lower limb muscles was not examined, and hence assessment of muscular atrophy was determined based only on examination of quadriceps muscle mass as well as detection of myosin levels indicative of the degree of myofibrils. Finally, only female mice were chosen for our current study due to the significantly easier post-operative care and lower risk of death following spinal cord injury. Hence, future animal models using both male and female mice would provide better gender diversity and reliability on the role of PTX in spinal cord injury. Despite these limitations, our findings provide novel insights into the neuroprotective mechanisms of PTX in SCI that further implicate its potential clinical application.

## Conclusions

Taken together, our findings elucidated that PTX prevents death of neurons and axons by decreasing neuronal apoptosis after SCI injury, via activating the Wnt/β-catenin signaling pathway. These findings also provide important insight and the theoretical basis for the potential use of PTX in the therapeutic treatment of spinal cord injury.

## Supplementary Information

Supplementary Material 1.

## Data Availability

No datasets were generated or analysed during the current study.
