# Supplementary material for: Paclitaxel alleviates spinal cord injury via activation of the Wnt/β-catenin signaling pathway
Source: Mol Med. 2025 May 6;31:172. doi: 10.1186/s10020-025-01240-3 (PMC12053863; doi:10.1186/s10020-025-01240-3)
Supplement: Supplementary file 1 — Supplementary Material 1. [file 10020_2025_1240_MOESM1_ESM.docx]

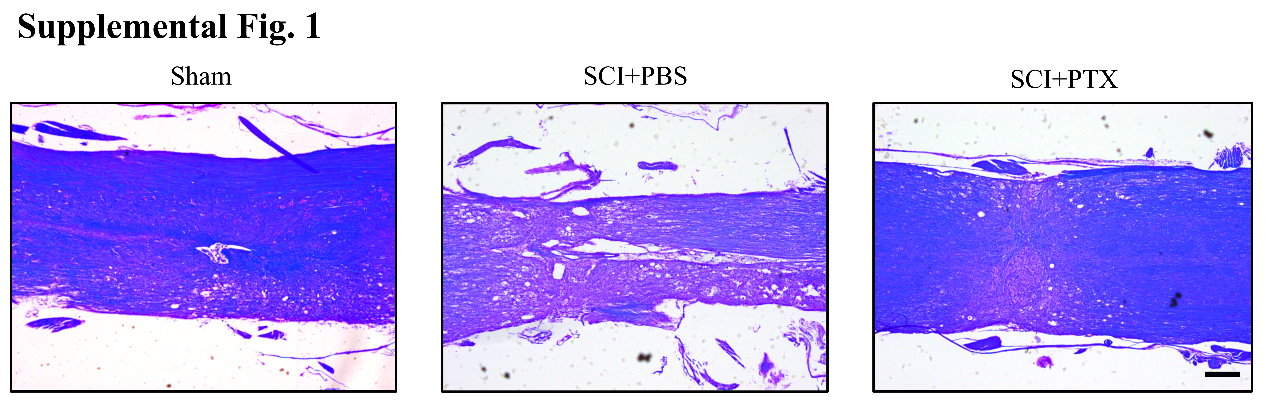


**Supplemental Fig. 1. PTX improves the morphology of damaged spinal cord tissue.** LFB staining results for the lengthwise section of the spinal cord in different groups at 21 days after SCI. Scale bar = 500 μm. n = 3 in each group.


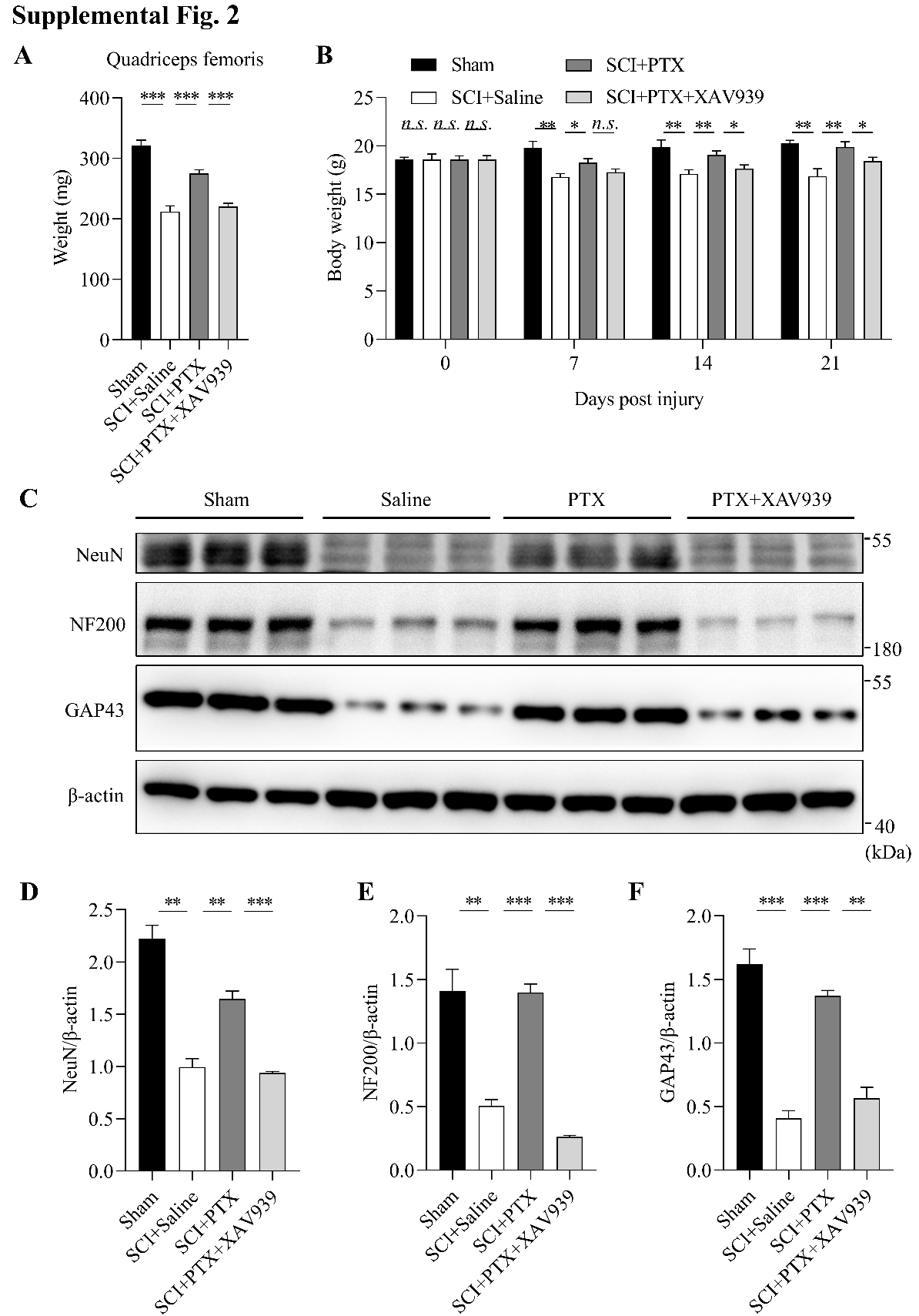


**Supplemental Fig. 2. Wnt pathway inhibitor XAV939 abolishes therapeutic effects of PTX after SCI.** (A and B) Body weight changes and quadriceps muscle weight in each group of mice on postoperative day 21. (C-F) Representative immunoblots and densitometry analysis of NeuN, NF200 and GAP43 expressions in the spinal cords of mice on postoperative day 21. β-actin, loading control. Data are presented as mean ± sem, **P* < 0.05, ***P* < 0.01, ****P* < 0.001. n = 3 or more in each group.
